# Root-Knot Disease Suppression in Eggplant Based on Three Growth Ages of *Ganoderma lucidum*

**DOI:** 10.3390/microorganisms10051068

**Published:** 2022-05-23

**Authors:** Saba Fatima, Faryad Khan, Mohd Asif, Saqer S. Alotaibi, Khushbu Islam, Mohammad Shariq, Arshad Khan, Mohd Ikram, Faheem Ahmad, Tabreiz Ahmad Khan, Rampratap Meena, Mansoor Ahmad Siddiqui

**Affiliations:** 1 Department of Botany, Aligarh Muslim University, Aligarh 202002, India; fatimasaba8272@gmail.com (S.F.); ansarishariq.amu@gmail.com (M.S.); arshad9758@gmail.com (A.K.); ikram.virologist@gmail.com (M.I.); tabreizkhan@gmail.com (T.A.K.); mansoor_bot@yahoo.co.in (M.A.S.); 2 Regional Ayurveda Research Institute, Central Council for Research in Ayurvedic Science, Ranikhet 263645, India; asifgc2616@gmail.com; 3 Department of Biotechnology, College of Science, Taif University, P.O. Box 11099, Taif 21944, Saudi Arabia; saqer@tu.edu.sa; 4 School of Life Sciences, Jawaharlal Nehru University, New Delhi 110067, India; khushbuislam1194@gmail.com; 5 Central Council for Research in Unani Medicine, New Delhi 110058, India; drpratapmeena@gmail.com

**Keywords:** biocontrol, egg hatching, *Ganoderma lucidum*, GC-MS, *Meloidogyne incognita*, mortality

## Abstract

This investigation presents a novel finding showing the effect of culture filtrates (CFs) of macrofungi, *Ganoderma lucidum*, against *Meloidogyne incognita* evaluated in vitro and in planta. To determine the nematicidal activity, juveniles of *M. incognita* were exposed to *Ganoderma* CFs of three different ages (Two, four and eight weeks old) of pileus and stipe at different concentrations, i.e., 100%, 50%, 10% and 1% for different time intervals (12, 24, 48 and 72 h). *Ganoderma* species were examined morphologically based on external appearance and analytically using SEM. The ethanolic samples of basidiocarp were prepared and analyzed for in vitro nematicidal assay and different bioactive compounds. The in vitro experiment results revealed that among all three ages of pileus and stipe, two-week-old pileus and stipe exhibited great nematotoxic potency and caused 83.8% and 73.8% juveniles’ mortality at 100% concentration after 72 h of exposure time, respectively. Similarly, the two-week-old pileus and stipe showed the highest egg hatching inhibition of 89.2% and 81.0% at the 100% concentration after five days. The eight-week-old pileus and stipe were not more effective than the two- and four-week-old pileus and stipe. The metabolites were characterized using GC-MS, including sugar alcohol, steroids, silanes, glucosides, pyrones, ester, oleic acid, phthalic acid, linoleic acid, palmitates and ketones. The in planta study conducted in the greenhouse demonstrated that the root dip treatment for 30 min with *Ganoderma* CFs curtailed the infection level of *M. incognita* and promoted the eggplant plant growth. The maximum percent increase in plant length, plant fresh weight, plant dry weight, total chlorophyll, carotenoids and yield/plant was obtained at 100% conc. of fungus CFs, whereas a reduction was observed in nematode infestation parameters. It was concluded from the study that *Ganoderma* CFs can be explored as an effective and eco-friendly antinemic biocontrol agent in fields infected with root-knot nematodes.

## 1. Introduction 

Eggplant is one of the most prevalent and economically important vegetable crops in India and elsewhere. This Solanaceae family member is cultivated throughout the world for its high nutritional importance. Eggplant ranks among the top ten vegetables having high antioxidants such as phenols and flavonoids [1]. The global production of eggplant in the year 2020 exceeded 56 million tons [2]. According to the FAO, in 2020, 94% of eggplant yield was produced by ten countries, namely, China, India, Egypt, Turkey, Iran, Indonesia, Italy, Japan, the Philippines and Spain. Eggplant is the most susceptible crop to common biotic stresses [3] and soil-borne pathogens or other pests may lead to 78% of the destruction of eggplant production if not tracked and managed properly [4].

Among these biotic stresses, phytoparasitic nematodes cause considerable damage and huge losses to a wide range of crops. The most dominating group of phytonematodes belongs to the genus *Meloidogyne*, a root-knot nematode. The *Meloidogyne* species are soil-borne pests that damage the host roots severely and get their nutrition by sucking up vascular tissues of the root, forming the giant cells. The physiology of the plant is badly disturbed; symptoms developed by the formation of root galls on the roots include reduced height of the plant and poor quality of the fruit developed. According to the All India Coordinated Research Project (AICRP), it was estimated that the loss of eggplant yield due to the economically important *Meloidogyne* species in India from 2014–2015 was 21% [5]. 

Plant-parasitic nematodes (PPNs) are the most difficult crop pests to control [6]. Unfortunately, due to their high rate of reproduction, it is not possible to eradicate PPNs from the field completely. We can only manage these field infections at the threshold level to protect the agronomy from huge damages. There are a number of management methods such as cultural practices, chemical methods, resistant varieties, organic amendment and biological control. Among them, the use of chemical nematicides are one of the primary means of control, but their incessant use makes the environment unhealthy and unsuitable for the survival of other living organisms. Therefore, there is a need to use a safer, cheaper and eco-friendly natural weapon against the *Meloidogyne incognita*. Biological management is one of the best strategies to control PPNs because antagonistic organisms have nematotoxic metabolites to reduce the nematode population. To date, fungi and bacteria remain the pre-eminent antagonists for PPNs biological control [7]. 

Macrofungi that are Basidiomycetes produce several bioactive compounds, such as polysaccharides [8], as well as have nematicidal properties in *Pleurotus* spp. [9]. *G. lucidum* or the mushroom of immortality is a basidiomycetous macrofungus. The genus *Ganoderma* consists of about 80 species and belongs to the family Ganodermataceae [10]. It is a popular medicinal mushroom that causes white rot disease on wood, which is why it is also called a wood-degrading fungus or decomposer. *G. lucidum* has a broad range of properties that make it unique from other fungi, such as being antibacterial, antifungal, antimicrobial, antiviral, etc. [11]. Several detailed studies and testing through HPLC, LCMS and GC-MS techniques were performed to explore the hidden qualities of bioactive macromolecules in *G. lucidum.* A group of research has been conducted on the basidiocarp, mycelia and spores of *G. lucidum* and explored the approximately 400 different bioactive compounds that exist in it [12]. Many studies of *G. lucidum* demonstrated its antibacterial nature and that its chemical constituents can provoke Gram-positive and Gram-negative bacteria [13,14]. An investigation showed the antifungal activities of ganodermin, a protein isolated from the fruiting body of *G. lucidum* [15], demonstrates its great potential to inhibit the growth of some harmful fungi such as *Botrytis cinerea*, *Physalospora oiricola* and *Fusarium oxysporum* [12]. Thus, *Ganoderma* continues to be frequently used to treat or inhibit a variety of disease causing pathogens [16,17,18,19]. However, there is no report yet on the nematicidal effect of *G. lucidum* on infected plants except two in vitro studies [20,21].

In the current study, the nematicidal ability of the macrofungus, *Ganoderma lucidum*, was evaluated in vitro and in planta as an eco-friendly approach for the management of *M. incognita*-infesting eggplant. In addition, an analysis of volatile organic compounds (VOCs) was carried out using the GC-MS technique. The present study highlights how effective are the extracts of different body parts (pileus and stipe) from different stages of *G. lucidum* at different concentrations against root-knot nematode, *M. incognita*. 

## 2. Materials and Methods

### 2.1. Collection, Identification and Microscopic Analysis of G. lucidum

**Figure 1:**
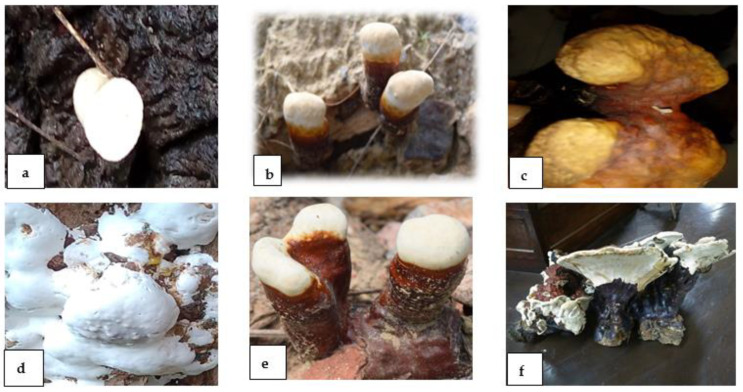
Morphology of different developmental ages, i.e., TW (two-week-old) (**a**,**d**), FW (four-week-old) (**b**,**e**) and EW (eight-week-old) (**c**,**f**) *G. lucidum*.

The macrofungus, *Ganoderma lucidum*, was grown in monsoon season (July to September) on the campus of Aligarh Muslim University (AMU), Aligarh. *Ganoderma* was collected from the campus of AMU and brought to the laboratory for further examination. The morphological identification of *G. lucidum* was based on the shape, color and size of the basidiocarp and basidiospores. The pileus or fruiting body is reniform, kidney or concave in shape and dark reddish-brown, whereas the stalk-like stipe or stem is cylindrical in shape and has a dark brown or brownish-black stalk. The species was found to be *G. lucidum* based on the presence of a brilliant or shiny surface of the fruiting body, which was confirmed by the morphological investigation conducted by Wang et al. [22] (Figure 1).

**Figure 2:**
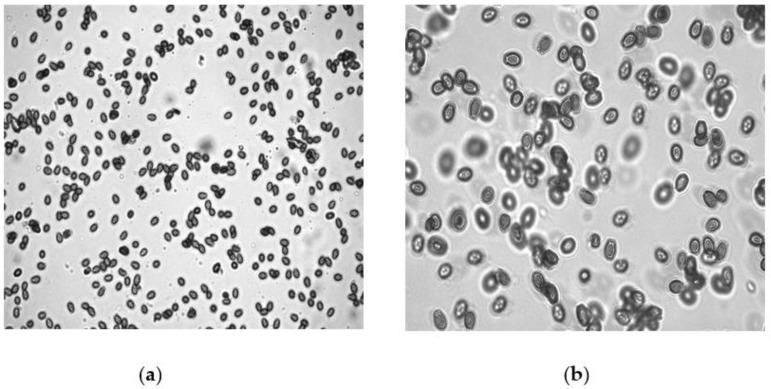
Inverted fluorescence microscope micrograph of basidiospores of *G. lucidum* at 40× and 100×. (**a**) 40× shows small, brown and ovoid basidiospores of *G. lucidum* (**b**) 100× shows that basidiospores appeared ovoid, truncated at the apex, with a dark brown eusporium bearing thick echinulae, surrounded by a hyaline myxosporium.

For further identification, the morphology of basidiospores was examined by following the technique of [23]. Basidiospores from the basidiome of *G. lucidum* were dusted on a clean microscopic slide and stained with 1–2 drops of lactophenol. The structure or shape of basidiospores was observed under an inverted fluorescence microscope (Leica DMi 8, Wetzlar, Germany) magnified at 40× and 100× (Figure 2a,b). Basidiospores were found to be small, ovoid, truncated at the apex and yellowish-brown with a dark brown eusporium bearing thick echinulae, surrounded by a hyaline myxosporium.

### 2.2. Scanning Electron Microscopy of Basidiocarp (Pileal Surface and Stipe) of G. lucidum

The ultrastructure of the basidiocarp of *G. lucidum* was analyzed using scanning electron microscopy (SEM, JSM 6510 LV, JEOL, Tokyo, Japan). Samples must be free from moisture to study the microstructure of hyphae of the pileus and stipe surfaces of *G. lucidum*. Samples were prepared using the method of Singh et al. [24]. Fresh *Ganoderma* was divided into two parts, i.e., the pileus and stipe. The pileus was then cut into small pieces 2 mm × 2 mm in size and the stipe was cut into small pieces of 4 mm × 4 mm. Specimens were fixed in a fixative (2.5% glutaraldehyde in 0.1% sodium phosphate buffer pH 7.2) for 24 h at 4 °C. Thereafter, samples were washed three times in a sodium phosphate buffer for 10–15 min in each wash. The second time, fixation was performed with 1% osmium tetraoxide for 2 h at 4 °C, followed by dehydration with 30% and 100% ethanol for 10 min. The samples were taken for CPD (critical point drying), and after CPD, dried specimens were mounted on an aluminum stub that had a piece of double-sided tape. Specimens were gold-coated (JEOL JFC-1600 Auto Fine Coater) with a layer about 14 nm thick [24]. The samples were now ready to be loaded into the SEM for basidiocarp surface viewing.

### 2.3. Cultivation and Maintenance of G. lucidum

The sample of the collected *Ganoderma* was inoculated into the three different heat-sealed cultivation bags, which contained substrate and had microfilter windows. The substrate of each bag consisted of rice bran (93 g), wheat bran (69 g), wood chips (1563 g) and water 775 g that resulted in a final moisture up to 55% in the mushroom bed. The temperature of the cultivated site was maintained at 23–25 °C, with air circulation for more than 12 h/day and humidity 80%. One sample of each developmental age, i.e., two-week-old (TW), four-week-old (FW) and eight-week-old (EW) *G. lucidum*, was harvested from separate bags [25].

### 2.4. GC-MS Analysis of G. lucidum

#### 2.4.1. Sample Preparation

For the analysis of the VOCs present in *G. lucidum*, a crude extract was prepared under laboratory conditions. The samples for the GC-MS analysis were prepared using the previously described method with slight modifications [26]. One hundred grams of basidiocarp (pileus and stipe separately) were washed with DW four times and air-dried on clean blotting sheets. The material was cut into small blocks and ground in a sterilized electric blender until fine. The grounded material (50 g) was soaked in a 1000 mL flask containing 500 mL of 100% ethanol. The mixture was kept for 72 h, then strained using a muslin cloth. The obtained decoction was centrifuged at 6000 rpm for 20 min, the pellet was discarded and the supernatant was filtered again using Whatman No. 1 filter paper. To avoid any contamination, the supernatant was treated with 2–3 drops of 0.1% streptomycin sulfate solution. Next, the filtrate was concentrated at 27 °C using a rotary evaporator to evaporate the ethanol. The resulting extract was stored at 4 °C in a glass vial until further use.

#### 2.4.2. Gas Chromatography-Mass Spectrometry (GC-MS) Conditions

The metabolites were detected and quantified by gas chromatography coupled with mass spectrometry (Shimadzu QP2010 Plus, Kyoto, Japan), equipped with a Rtx-5 MS capillary column (30 m in length, internal diameter and film thickness each 0.25 mm). The derivatization procedure was performed using N-Methyl-N-(trimethylsilyl) trifluoroacetamide (MSTFA). A sample volume of 250 µL was transferred into GC glass vials and 1 µL was injected into the column with a split ratio of 10 at the rate of 1.21 mL per minute using helium as a carrier gas. The temperature of the oven was set at 100 °C, then increased to 250 °C by an increment of 5 °C per min and, finally, to 280 °C by an increment of 10 °C per min. Distinct peak fragmentation patterns of metabolites were detected using an MS detector in a full scan mode. The data were analyzed using the software GCMS Solutions (Lab Solutions ver. 2.5 Shimadzu, Kyoto, Japan); the peaks were integrated manually and the chromatograms were deconvoluted in the same way. The identification of metabolites was performed based on the retention times. The quantification of various metabolites was performed using their respective peak areas and the molecular masses were obtained. The metabolites identified were confirmed by comparing the peak spectra with standard mass spectra from three library databases, including the National Institute of Standards and Technology’s NIST 14 and NIST 14s (https://www.nist.gov/, accessed on 28 February 2022) and Wiley 8. The metabolite compounds were normalized against the internal standards.

### 2.5. Inoculums Maintenance and Species Identification of M. incognita

**Figure 3:**
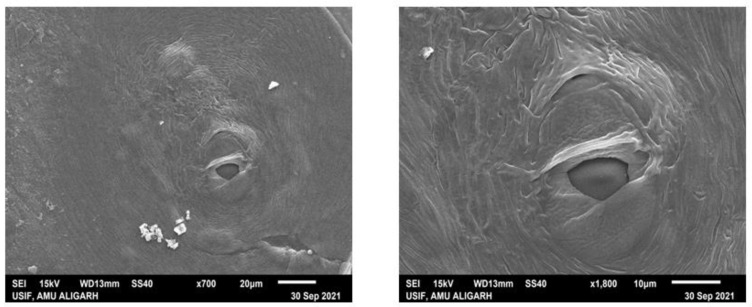
SEM micrograph of perineal pattern of *Meloidogyne incognita* at different magnifications.

The pure population of root-knot nematodes (RKNs), *Meloidogyne incognita*, was maintained and cultured on eggplant plants under greenhouse conditions (temperature 27 ± 2 °C, adequate photoperiod of 11 ± 1 h and humidity 65% to 80%) at the Department of Botany, AMU, Aligarh (India). Scanning electron microscopy (SEM) was utilized for species identification of the root-knot nematode. The sample was prepared using the perineal pattern technique given by [27]. A healthy female was isolated from the infected eggplant root and placed on a clean slide under a stereoscopic microscope. The section of female was cut in such a manner that the posterior portion exhibited a perineal pattern that was completely visible. Three sharp cuts were made on the posterior side of the female body; the first cut was made transversely and two cuts were made longitudinally, with one on the left and the other on the right side. The specimen was cleaned and put in 1–2 drops of lactophenol. The specimen was transferred to a new slide and kept in different series of ethanol (70%, 90% and 100% ethanol). Further steps such as CPD and the mounting and coating of samples were performed in a similar manner, using scanning electron microscopy as described above. The presence of a high dorsal arch and wavy lines, key features of *Meloidogyne incognita*, were observed (Figure 3). 

### 2.6. Inoculum Preparation of M. incognita Juveniles

Infected roots were washed delicately to free them from adhering soil. The egg masses were handpicked and collected into coarse sieves, which were already layered with tissue paper, then kept in a petri plate containing DW. The petri plates were incubated at 27 ± 2 °C in the BOD incubator. After every 2 days, the juveniles’ suspension was collected and freshwater was added. The collected juveniles’ suspension was standardized as per requirement for in vitro and in planta experiments. 

### 2.7. Toxicity Testing of G. lucidum against M. incognita Juveniles: In Vitro

#### 2.7.1. Preparation of Culture Filtrates (CFs) of *G. lucidum*

Culture filtrates of each age of the basidiocarp (pileus and stipe separately) of *G. lucidum* were prepared for in vitro testing. *G. lucidum* was surface sterilized with 0.1% of NaOCl and washed gently with distilled water (DW) four times. The cleaned pileus and stipe were placed on blotting sheets for drying at room temperature. The pileus and stipe were cut into small pieces and both materials were ground separately with the help of an electric blender. An amount of 50 g of each part was transferred into a 1 L flask containing 1000 mL of distilled water and left for 24 h. The mixture of fungus was filtered with the help of a cheese cloth, followed by Whatman No. 1 filter papers. The filtrate was centrifuged at 6000 rpm for 20 min to obtain a clear solution and was considered as stock for further use.

The stock solution of the pileus and stipe of each stage of *G. lucidum* was considered as standard or as a treatment of 100% concentration. This standard concentration was diluted with DW to obtain 50%, 10% and 1% concentrations. Two drops of 0.1% streptomycin sulfate solution was added to avoid bacterial contamination.

#### 2.7.2. Inhibition in Hatching of *M. incognita* Eggs Exposed to the CFs of *G. lucidum*

For hatching purposes, five fresh and healthy egg masses were transferred into each petri dish containing 5 mL of CF of all three ages with different concentrations (100%, 50%, 10% and 1%) of the pileus and stipe of the *Ganoderma*. All petri dishes were kept in an incubator at 28 ± 2 °C for 5 days. The total number of hatched second-stage juveniles (J2) was counted in a counting dish under a stereoscopic microscope.

#### 2.7.3. Mortality and Immobility of J2 of *M. incognita* Exposed to the CFs of *G. lucidum*

The culture filtrate (4.8 mL) of each dilution of TW-, FW- and EW-old pileus and stipe extracts was put into petri dishes. An amount of 0.2 mL of pure suspension of *M. incognita* containing one hundred freshly hatched juveniles was added to the treated and control petri dishes, in which the latter had only DW. All the petri dishes were kept on a laboratory bench at room temperature and the total number of dead juveniles was counted using a counting dish after 12, 24, 48 and 72 h of exposure time under the stereo microscope. According to El-Rokiek and El-Nagdi, [28], if there is any movement and the shape of the nematode is winding, then it is considered as alive. The nematode is found dead when there is no movement or it is straight. Keeping these points in mind, the number of both alive and dead nematodes was recorded. The percent mortality or inhibition in egg hatching was calculated by using the formula:

% inhibition or mortality = {(C0 − Tα)/C0} × 100

where

in the case of hatching,

C0 = number of juveniles hatched in the control and Tα = number of juveniles hatched in each concentration of CF.

In the case of mortality, 

C0 = number of alive nematodes in the control and Tα = number of alive nematodes after the 12, 24, 48 and 72 h exposure period.

### 2.8. SEM of Treated Juveniles (TJ) and Untreated Juveniles (UTJ) of M. incognita

To explore the impact of *G. lucidum* on the external surface of the root-knot nematode, *M. incognita*, juveniles were treated with the CF of the TW-old pileus of *G. lucidum*. Specimens from treated and control petri dishes (healthy juveniles) were handpicked with the help of a horse tail hair attached to a needle and fixed in 40% formaldehyde for 24 h. Fixed specimens were transferred into 3% glutaraldehyde [29] for 48 h. After that, specimens were transferred to capsules and washed with a 0.05 M sodium phosphate buffer twice. The specimens were dehydrated in a graded ethanolic series (30, 50, 70, 80, 90 and 100%) and carried for CPD (critical point drying) in carbon dioxide [27]. Dried specimens were kept in a desiccator and mounted on stubs, coated with gold. The nematode surface was analyzed after loading the specimens in the SEM (model, JOEL JSM 6510 LV) at a different resolution at 15 KV. 

### 2.9. In Planta Study (Pot Experiment)

The pot study was conducted under greenhouse conditions. Three-week-old healthy eggplant seedlings were exposed to the CFs of two-week-old *Ganoderma* (100% conc.), which were applied as a root dipping treatment for 30 min along with the control. Afterward, all the seedlings were transferred in autoclaved earthen pots (30 cm diameter) filled with 2 kg of sandy loam soil and manure in the ratio of 3:1. Each treatment was replicated five times, including the control (untreated uninoculated control, UUC). Three days later, each plant was inoculated with 3000 freshly hatched juveniles (J2) of *M. incognita* by making three holes around the soil without damaging the roots. The plants were watered and cared for time to time. 

### 2.10. Data Collection and Observations

Three months after inoculation, all plants were uprooted gently and washed in a bucket filled with water to remove adhering soil. Care was taken while dealing with roots to ensure the safety of root galls. The data for plant growth were analyzed in terms of shoot and root fresh weight, shoot and root dry weight, yield and physiological (total chlorophyll and carotenoids) and pathological parameters (juveniles/kg soil, females/root system, no. of galls/root system and number of eggs/egg-mass). Daykin and Hussey’s [30] methodology was adopted for examining egg masses. Cobb’s sieving and decanting procedure [31] was used to determine the total population of the nematodes, followed by a modified Baermann funnel technique [32]. The total amount of chlorophyll and carotenoids in the fresh leaf tissues was analyzed following the protocol given by Mackinney [33]. The following formula was used to determine the chlorophyll and carotenoid content of the sample (mg/g fresh leaf).

Total chlorophyll = {20.2(A_645_) + 8.02(A_663_)} × (V/1000 × W).

Carotenoid = {7.6(A_480_) − 1.49(A_510_)} × (V/1000 × W).

A_480,_ A_510,_ A_645,_ A_663_ = absorbance of extract at given wavelengths (480, 510, 645, and 663 nm, respectively).

V = final volume of the extract(1)

W = fresh weight of the leaf sample(2)

D = length of the path of light(3)

### 2.11. Statistical Analysis

Each treatment was conducted in five replications. Data on hatching and mortality were displayed in terms of values of percent mortality over the control. Experimental data from the pots study were analyzed by a one-way analysis of variance (ANOVA) using the SPSS-17.0 statistical software (SPSS Inc., Chicago, IL, USA). The variability between mean values of treatments was determined by Duncan’s multiple range test (DMRT) at *p* ≤ 0.05.

## 3. Results

**Figure 4:**
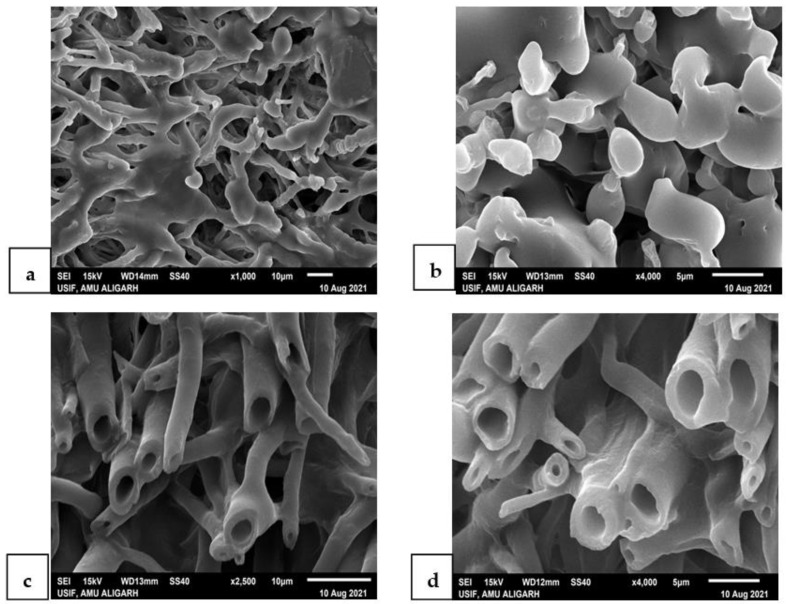
Scanning electron microscopy (SEM) micrograph of (**a**,**b**) upper body (pileus) and (**c**,**d**) lower body (stipe) of *G. lucidum*.

The morphological changes of the fruiting bodies of *G. lucidum* during its development were categorized into three successive stages or ages, as can be seen in Figure 1. The scanning electron microscopic analysis of the pileus and stipe and inverted fluorescence microscopic investigation of the spores of the fruiting body of *Ganoderma* gave conformity to the species of *G. lucidum* (Figure 2 and Figure 4). Basidiospores appeared small, ovoid, truncated at the apex and yellowish-brown with a dark brown eusporium bearing thick echinulae, surrounded by a hyaline myxosporium. Perineal pattern identification via scanning electron microscopy provided confirmation about the species of *Meloidogyne incognita*, showing the presence of a high dorsal arch with wavy lines. For the in vitro test, four variable concentrations (100%, 50%, 10% and 1%) of CFs of both parts, i.e., the pileus and stipe, were taken as treatments, along with DW as the control. The ascendancy of different treatments of CFs of two isolated parts (pileus and stipe) of *G. lucidum* acted differently on the hatching and mortality of the second-stage (J2) juveniles of *M. incognita* at different exposure times (12, 24, 48 and 72 h). The evaluation of the toxicity of pileus and stipe CFs of *G. lucidum* on the hatching and mortality of J2 of *M. incognita* was found significant.

### 3.1. Effect of Different Concentrations of G. lucidum CFs on the Egg Hatching of M. incognita

It is apparent by the perusal of data presented in Table 1 and Table 2 that after five days of incubation the culture filtrate of the pileus and stipe of *G. lucidum* showed the nematicidal effect to varying degrees on egg hatching of the root-knot nematode, *M. incognita.* At 100% concentration, the highest inhibition in the emergence of *M. incognita* juveniles was recorded in TW-old pileus CFs, followed by TW stipe, FW pileus, FW stipe, EW pileus and EW-old stipe CFs. The corresponding percentage of suppression in egg hatching was recorded as 89.2, 81.0, 77.1, 69.1, 55.9, and 45.0%, as compared to the control (0.0%). Similarly, at 50% conc., inhibition in the emergence of juveniles from the eggs in respective CFs was observed as 72.8, 68.5, 51.0, 34.0, 39.0, and 31.0%, as compared to the control (Table 1 and Table 2).

In the 10% treatment, the greatest reduction in egg hatching was noticed in TW-old pileus CF (53.0%), followed by TW stipe (42.3%), FW pileus (33.1%), FW stipe (25.1%), EW pileus (29.9%) and EW stipe (22.3%), as compared to the control (0.0%). The least inhibition in egg hatching was found at 1% conc. and was recorded as 24.0, 20.0, 18.7, 14.9, 14.0 and 7.6% in the corresponding CFs of different parts of *G. lucidum* (Table 1 and Table 2). However, there was no significant difference in the inhibition of egg hatching noticed in the 1% treatment of FW- and EW-old stipe CFs.

There was a relatively significant decrease in the eggs hatching with the corresponding increase in the concentration of fungus CFs. Inhibition in egg hatching at different treatments significantly declined with the increase in the age of the parts of *G. lucidum*. Among the different parts (pileus and stipe) of *G. lucidum*, the pileus CFs showed the most significant and greatest inhibition in the eggs hatching as compared to the stipe of the fungus.

### 3.2. Effect of Different Concentrations of G. lucidum CFs on the Mortality of M. incognita

The data presented in Table 3 and Table 4 indicated that the mortality of juveniles of *M. incognita* was observed to be significant (*p* ≤ 0.05) at different concentrations of pileus and stipe CFs at different exposure periods. The percent mortality of J2 was directly proportional to the concentration of fungal extracts of pileus and stipe and the period for which the nematode was exposed to the extract. No percent mortality was recorded in all the treatments of *G. lucidum*, even after 12 h of the exposure period. For the pileus, 83.8% mortality was found at a 72-h exposure time in 100% concentration of CFs of two-week-old (TW) *Ganoderma.* Certainly, the CFs of the pileus was more efficacious in killing juveniles of *M. incognita* than CFs of the stipe. TW pileus in 100% treatment for 72 h of incubation occupied the supremacy of the reduction in nematode juveniles over other TW-, FW- and EW-old treatments (Table 3). The rate of mortality was low in the beginning, but an appreciable increase was found only after 24 h of the exposure. Mortality of juveniles reached up to 67% on FW-old pileus in 100% treatment for a 72-h exposure period. The least amount of mortality of 47% was found on EW-old pileus in the 100% treatment for a 72-h incubation period, compared to 0% in the control. 

A nearly similar trend of mortality was recorded for the set-ups treated with CFs of different ages of the stipe (Table 4). The highest mortality (73.8%) was seen in TW-old stipe with the 100% treatment and a 72-h exposure time. The FW- and EW-old stipe showed 61.6% and 38.9% of J2 mortality in the 100% treatment with a 72-h exposure period, respectively. After 12 h of the exposure period, there was no percent mortality observed in the EW-old pileus- and stipe-treated nematodes in all concentrations. The mortality of juveniles of *M. incognita* was found nil in all the 1% treatments of TW-, FW- and EW-old pileus and stipe at 12 h. In most of the treatment concentrations and exposure periods, the mortality of *M. incognita* was significantly decreased with the increase in age of *G. lucidum*.

### 3.3. GC-MS Analysis for Bioactive Compounds

**Figure 5:**
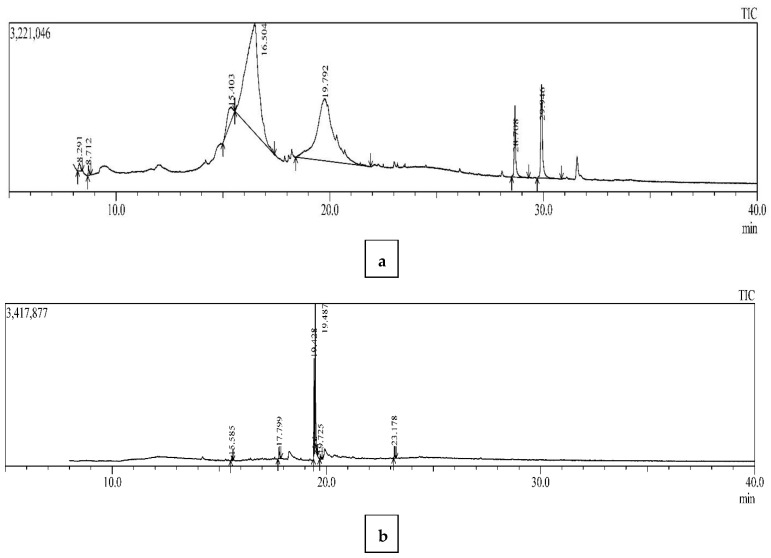
GC-MS chromatogram showing peaks of bioactive compounds obtained from ethanolic extract of *G. lucidum* parts: (**a**) pileus and (**b**) stipe.

A total of 13 metabolites were identified in the basidiocarp (pileus and stipe) of the *G. lucidum*. The results revealed different bioactive compounds; seven in the pileus and six in the stipe of the *G. lucidum* were detected. The GC-MS analysis showed that the metabolites obtained belonged to diverse chemical functional groups, including pyrones (2,3—Dihydro—3,5—Dihydroxy—6—Methyl—(4H)—pyran—4—one), alkenes (2—Hexene—3,4,4—Trimethyl), glucosides (Beta—D—Glucopyranoside, methyl), sugar alcohols (DL—Arabinitol and D—Mannitol), silanes (Silane, dimethyl (3—fluorophenoxy) tetradecyloxy) and steroids (Cholesta—8,14—dien—3—ol, 3 beta, 5—alpha) present in the pileus (Table 5, Figure 5a), and cyclic ketone ((3aR, 4R, 7R)—1,4,9,9—Tetramethy l—3,4,5,6,7,8—hexahydro—2H—3a, 7—methanoazulen—2—one), palmitates (hexadecanoic acid, methyl ester), linoleic acids (9,12—Octadecadienoic acid (Z, Z)-, methyl ester), oleic acids (9—Octadecenoic acid, methyl ester, (E), Ester (Methyl stearate) and Phthalic acid (Bis (2—ethylhexyl) phthalate) present in the stipe of *G. lucidum* (Table 5, Figure 5b). The important information, such as the name of the compound, retention time, peak area, peak area (%), chemical formula and chemical functional group, were listed in Table 5.

### 3.4. SEM of the Treated Juveniles (TJ) and Untreated Juveniles (UTJ)

**Figure 6:**
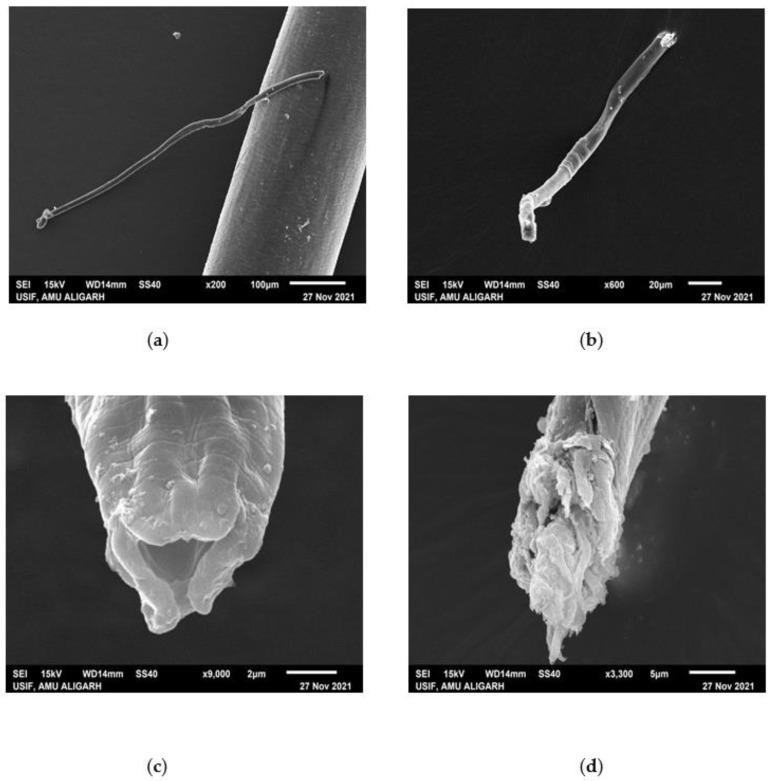
SEM micrograph of untreated (**a**,**c**) and treated (**b**,**d**) *Meloidogyne incognita*. Untreated (**a**,**c**) shows no splitting and no deformation in the body of the nematode, whereas (**b**,**d**) when treated with *G. lucidum* for 3 days, clear deformation was found on the body of the nematode.

SEM analysis of the TJ and UTJ showed a great difference in their surface morphology. Figure 6 indicated that treated juveniles were deformed in their structure, appearance and size as compared to the untreated control. As we can see in Figure 6, the entire body of the UTJ (a) was found as it is, whereas the TJ (6b) was missing the anterior portion. In comparison, a high magnification view of the anterior side depicts that there was a huge disruption of the mouth region in the TJ (6c), whereas the anterior shape was perfect in the UTJ (6d). These morphological alterations in TJ specimens were due to the toxicity of the culture filtrate of *G. lucidum*. The GC-MS identified some metabolites that have a nematotoxic effect on the morphology of the nematode. 

### 3.5. Effect of Root Dip Treatment with CFs of G. lucidum on Multiplication of M. incognita Parasitized Eggplant (in Planta Study)

The data analysis of the in vitro test on hatching and mortality of *M. incognita* revealed that CFs of *G. lucidum* can curtail the population of nematodes. To examine whether the root dip treatment of *Ganoderma* CFs affects *M. incognita* parasitism during a compatible interaction, a pot study was conducted where the CFs of TW-old *Ganoderma* were applied as a root dip treatment on eggplant susceptible to *M. incognita*. Significantly, the highest decline in the number of galls (68), number of egg masses (97), number of eggs/egg mass (129), juveniles/kg soil (5293) and adult females per root system (123) was achieved when roots of healthy seedlings were dipped in 100% CFs of *G. lucidum* for 30 min (Table 6). The lowest reduction in nematode infestation was observed at 10 and 1% concentrations with the minimum number of galls (126 and 132), the number of egg masses (131 and 137), the number of eggs per egg mass (330 and 342), juveniles/kg soil (11,942 and 12,349) and females/root system (259 and 278), respectively (Table 6). The values of pathological parameters for 10 and 1% treatments were closely related to values of the untreated inoculated (UIC) control, which exhibit the highest number of galls (139), number of egg masses (146), number of eggs/egg mass (355), juveniles/kg soil (12,844) and females/root system (327) (Table 6). The concentrated form of *Ganoderma* CFs with 100% conc. significantly suppressed the infection intensity of *M. incognita* as compared to the UIC. 

### 3.6. Effects of Root Dip Treatment with CFs of G. lucidum on Eggplant Growth

Treatments of *Ganoderma* CFs as a root dip for 30 min significantly enhanced the plant growth and yield of eggplant. The treatment (100%) improved the highest plant growth in terms of shoot length (48.39 cm), root length (18.86 cm), shoot fresh weight (220 g), root fresh weight (92.0 g), shoot dry weight (34.20 g), root dry weight (10.45 g) and yield per plant (605) compared to the control (UIC), followed by the treatment of 50% that presented the second-best result of promoting plant growth, shown in Table 7. The UIC gave the lowest growth for the shoot (25.45 cm) and root (13.3 cm) length, shoot (110 g) and root (40.0 g) fresh weights, shoot (22.63 g) and root (6.45 g) dry weights and yield (347). The diluted forms of CFs of fungus, i.e., 10 and 1%, resulted in slightly improved plant growth compared to the UIC. The treatment of 10% conc. showed better growth than the UIC in terms of shoot length (45.32 cm), root length (17.25 cm), shoot fresh weight (147 g), root fresh weight (61.0 g), shoot dry weight (23.50 g), root dry weight (7.40 g), and yield per plant (432) (Table 7). The smallest growth among all the concentrations of *Ganoderma* was recorded in the 1% treatment, with shoot length, root length, shoot fresh weight, root fresh weight, shoot dry weight, root dry weight and yield per plant having the corresponding values of 43.56 cm, 16.99 cm, 132 g, 48 g, 22.10 g, 6.40 g and 376, respectively. The values of plant growth of the 1% treatment were not equal to, but were almost the same as the growth values of the untreated inoculated control (Table 7). The data presented in Table 7 depicted that the best significant growth promotion was observed in the 100% treatment compared to the untreated uninoculated control (UUC).

### 3.7. Effect of CFs of G. lucidum on the Physiology of Eggplant

Similarly, the use of *G. lucidum* CFs as a root dip treatment for 30 min stimulated the physiological activity of the plant. The highest content of total chlorophyll (1.93 mg/g) and carotenoids (0.81 mg/g) was observed in the 100% treatment of *G. lucidum* (Table 7). On the other hand, the lowest value of total chlorophyll and carotenoids, i.e., 1.49 mg/g and 0.58 mg/g, respectively, were found in the 1% concentration of the *Ganoderma*. The values of the 1% treatment were slightly greater than the values of the UIC, which were 1.45 for chlorophyll and 0.54 for carotenoids (Table 7).

## 4. Discussion

The motive of the current investigation was to deduce important information for the effective formulation of the king of the herb, ‘*G. lucidum*’. This macrofungus was utilized at three different ages to reduce the disease-causing intensity of the root-knot nematode, *M. incognita*. Among the different developmental stages of the root-knot nematode, second-stage juvenile (J2) is the infective stage where it hatches out and searches for a loving host to penetrate root tissues. They obtain their nourishment by destroying the root system of the host plant (eggplant). To overcome this problem, we tested the impact of different concentrations of CFs of TW-, FW- and EW-old *G. lucidum* on the egg hatching and mortality of *M. incognita*. It was clear from the results that after five days, different treatments of *G. lucidum* CFs showed inhibition in the hatching of eggs of *M. incognita* to a varying degree, irrespective of the age of *G. lucidum*. There was a relatively significant decrease in the number of eggs hatching that corresponded with an increase in the concentration of fungus CFs. Inhibition in egg hatching with different treatments significantly decreased with the increase in the age of *G. lucidum*, except with the 1% treatment of four- and eight-week-old stipe. The pileus of *G. lucidum* CFs showed significantly the greatest inhibition in egg hatching as compared to the stipe of the fungus. In the “100%” treatment, the highest inhibition in the juvenile emergence of *M. incognita* was recorded in the two-week-old pileus CF, followed by two-week stipe, four-week pileus, four-week stipe, eight-week pileus and eight-week-old stipe CFs.

Similarly, the extracts of pileus and stipe of *G. lucidum* manifest the nematicidal effect to varying degrees on *M. incognita*. The percentage of mortality of nematodes was directly proportional to the treatment of fungal CFs and the period for which the nematode was exposed to the extract. In most of the concentrations of the treatment and exposure periods, the mortality of *M. incognita* significantly decreased with the increase in age of *G. lucidum*. Generally, the pileus CF showed significantly the greatest mortality of juveniles as compared to the stipe extract of *G. lucidum* in different concentrations and exposure periods. The nematicidal potential of the extract of fungus on the plant-parasitic nematode may be attributed to the production of certain toxic metabolites, such as triterpenes, sesquiterpenes, applannoxidic acid, steroids and bioactive peptidoglycans by *Ganoderma* species [34,35,36]. Akshaya et al. [20] and Themuhi et al. [21] observed 93.2% juvenile mortality and 92.6% egg hatching inhibition at a 1000 ppm concentration of *G. lucidum* extract. These results are also in agreement with the findings of Bua-art et al. [37] in which the bioactive compound extracted from *Neonothopanus nambi* at a concentration of 500 mg/L caused 100% mortality of J2 of *Meloidogyne incognita* in 1 min. The concentrations of 100 and 50 mg/L affected the J2 and resulted in a significant mortality of 100% in 30 min and 48 h, respectively. The effect of the bioactive compound on J2 was also confirmed in a screened-house experiment and found that concentrations of 100 and 500 mg/l suppressed J2 without root-knot symptoms on tomato plants. The adverse effect of the culture filtrate of several other fungi on the mortality and hatching of nematodes has been reported by other workers also [38,39,40,41].

The variable effect of pileus and stipe CFs on the hatching and mortality of root-knot nematodes observed in the present studies can be attributed to the varied nature of the toxic metabolites produced by *G. lucidum* in different parts of the fruiting body. GC-MS analysis of the pileus and stipe of *G. lucidum* explored thirteen different bioactive compounds that belong to diverse groups of secondary metabolites, including pyrones, glucosides, sugar alcohols, silanes and steroids present in the pileus, and cyclic ketone, palmitates, linoleic acids, oleic acids, ester and phthalic acid present in the stipe of *G. lucidum*. It can be concluded that the presence of bioactive compounds is responsible for curtailing the nematode population effectively. The antimicrobial properties of *G. lucidum* were found effective in inhibiting the development of nematodes. The surface investigation of the TJ and UTJ using scanning electron microscopy provided confirmation on the lethal effect of *G. lucidum* toxicity against nematodes. It was crystal clear from comparing the TJ with the UTJ that the extract of *G. lucidum* interferes with the metabolic activities of nematodes, resulting in the degradation of juveniles.

There are only two findings available regarding the nematicidal potential of *Ganoderma lucidum* [20,21], in which they investigated the inhibitory potential of an ethyl acetate extract of *Ganoderma* lucidum and *Lentinus edodes* on *M. incognita*. Akshaya et al. [20] also showed twenty-two bioactive metabolites of *G. lucidum* by GC-MS analysis. In the present study, it was noted that the three bioactive compounds of the pileus ethanolic extract, including hexadecanoic acid, methyl ester; methyl stearate and Bis (2—Ethylhexyl) Phthalate, resembled the same compounds identified already by investigators Akshaya et al. [20]. Zhao et al. [42] explored the toxicity of bioactive compounds released by *Simplicillium chinense* Snef5 on the suppression of the root-knot nematode, *M. incognita.* Limited research has been conducted on the fungicidal activity of it. The adverse effect of *G. lucidum* against different fungi has also been reported by Innocenti et al. [43]. Badalyan et al. [44] reported the antagonistic activity of 17 species (*C. versicolor*, *F. velutipes*, *G. lucidum*, *H. fasciculare*, *H. sublateritium*, *K. mutabilis*, *L. edodes*, *P. alnicola*, *P. aurivella*, *P. destruens*, *P. ostreatus*, *P. cornucopiae*, *P. squamosus*, *P. subarcularius*, *P. varius* and *S. commune*) against four fungi (*B. sorokiniana*, *F. culmorum*, *G. graminis var. tritici* and *R. cerealis*), responsible for foot and root disease of cereals. Almost all tested mushroom species markedly inhibited mycelia growth of the four phytopathogenic fungi; however, the antagonistic activity of *P. ostreatus*, *G. lucidum*, *H. fasciculare*, *L. tigrinus* and *S. commune* was stronger. Sridhar et al. [45] evaluated the antifungal activity of the fruiting body of *Ganoderma lucidum* against five fungi, viz., *Penicillium* spp., *Aspergillus fumigatous*, *A. niger*, *A. flavus* and *Mucor indicus*. For antifungal activity, the zone of inhibition of microorganisms was measured in mm. The methanolic extract showed the maximum antifungal activity and a 30 mm inhibition zone was recorded in 200 mg of the extract against *Mucor indicus*, whereas a minimum 3 mm inhibition zone was recorded in 50 mg of the extract against *Aspergillus flavus.* Liu et al. [46] also conducted studies on various nematophagous fungi.

There is no pot study available regarding plant disease management to manage or control *Meloidogyne incognita* in greenhouse or field. In the present study, *G. lucidum* was used as a root dip treatment for the first time in the greenhouse condition. The data analysis indicates that the TW-old *G. lucidum* significantly regulated growth and physiology in terms of fresh shoot and root weights, dry shoot and root weights and chlorophyll and carotenoid content of eggplant plants. There was a great increase in yield per plant. *Ganoderma lucidum* showed a significantly negative correlation towards nematode development. As the concentration of *G. lucidum* increased, there was a significant decrease in juvenile numbers, adult females, number of galls and number of egg masses.

In the present investigation, the extract of *G. lucidum* could be used as a biological control for the management of nematodes. The interest of scientists can adhere to the development of novel nematicidal/fungicidal compounds derived from fungal culture filtrates. These toxic fungus metabolites can effectively target the nematodes/fungi and are less hazardous to the environment than traditional pest control chemical methods. Due to its significant nematicidal activity shown under laboratory and greenhouse conditions, the *G. lucidum* CFs may be tested for their ability to control *M. incognita* under field conditions. More research can be conducted to isolate and identify the effective components present in fungal extract which are responsible for their pesticidal activity. 

## 5. Conclusions

The fungus can colonize a plant’s roots and secrete secondary metabolites against pathogens and stimulate plant growth. The entry of the phytopathogens into the root can be inhibited by the interference of metabolites secreted by biocontrol agents, including the root-knot nematodes. The present investigation revealed that the production of different bioactive compounds by *Ganoderma lucidum* has a toxic effect on *M. incognita* juveniles, inhibiting egg hatching and reducing the nematode population. Two-week-old pileus and stipe were found most effective against the nematode activity. SEM analysis of *Ganoderma*-treated juveniles indicated that the compounds present in *Ganoderma* significantly degraded the entire body of the nematode. It was concluded that the bioactive compounds released from *G. lucidum* can be used as a promising biocontrol product or TW-old *G. lucidum* as a biocontrol agent for future root-knot nematode management strategies, and also provide an excellent alternative approach in place of nematicides.

## Figures and Tables

**Table 1:** Effect of different stages of pileus of *Ganoderma lucidum* on egg hatching inhibition of root-knot nematode, *Meloidogyne incognita*.

Age of Fungus	Concentration (%)	Inhibition (%)
Two week	100	58 (89.2) ^a^
	50	146 (72.8) ^b^
	10	253 (53.0) ^c^
	1	409 (24.0) ^f^
Four week	100	123 (77.1) ^b^
	50	264 (51.0) ^c^
	10	360 (33.1) ^e^
	1	437(18.7) ^g^
Eight week	100	237 (55.9) ^c^
	50	328 (39.0) ^d^
	10	377 (29.9) ^e^
	1	462 (14.0) ^h^
Control	-	538 (0.0) ^i^

Each value is the mean of five replicates; values in parentheses are percent inhibition over control; values in each column followed by the same in the letter are not significantly different according to Duncan’s multiple range test (DMRT) at *p* ≤ 0.05.

**Table 2:** Effect of different stages of stipe of *Ganoderma lucidum* on egg hatching inhibition of root-knot nematode, *Meloidogyne incognita*.

Age of Fungus	Concentration (%)	Inhibition (%)
Two week	100	102 (81.0) ^a^
	50	169 (68.5) ^b^
	10	312 (42.3) ^c^
	1	430 (20.0) ^f^
Four week	100	166 (69.1) ^b^
	50	355 (34.0) ^d^
	10	403 (25.1) ^e^
	1	458 (14.9) ^g^
Eight week	100	296 (45.0) ^c^
	50	371 (31.0) ^d^
	10	418 (22.3) ^f^
	1	497 (7.6) ^h^
Control	-	538 (0.0) ^i^

Each value is the mean of five replicates; values in parentheses are percent inhibition over control; values in each column followed by the same in the latter are not significantly different according to Duncan’s multiple range test (DMRT) at *p* ≤ 0.05.

**Table 3:** Effect of different stages of pileus of *Ganoderma lucidum* on juvenile mortality of *Meloidogyne incognita*.

Age of Fungus	Exposure Period (h)	Concentration (%)	Juvenile Mortality (%)
Two week	12	100	25.9 ^j^
		50	18.9 ^n^
		10	12.2
		1	0.0 ^w^
	24	100	40.5 ^f^
		50	28.6 ^i^
		10	19.3 ^mn^
		1	8.4 ^stu^
	48	100	58.6 ^c^
		50	36.1 ^g^
		10	23.6 ^kl^
		1	8.1 ^tu^
	72	100	83.8 ^a^
		50	54.3 ^d^
		10	36.3 ^g^
		1	20.9 ^mn^
Four week	12	100	19.5 ^mn^
		50	11.4 ^qr^
		10	0.0 ^w^
		1	0.0 ^w^
	24	100	24.4 ^kl^
		50	16.5 ^op^
		10	7.0 ^u^
		1	0.0 ^w^
	48	100	39.9 ^d^
		50	27.7 ^ij^
		10	15.9 ^p^
		1	3.9 ^v^
	72	100	67.3 ^b^
		50	39.0 ^f^
		10	29.0 ^i^
		1	15.0 ^p^
Eight week	12	100	0.0 ^w^
		50	0.0 ^w^
		10	0.0 ^w^
		1	0.0 ^w^
	24	100	16.7 ^op^
		50	9.1 ^rst^
		10	2.1 ^vw^
		1	0.0 ^w^
	48	100	29.2 ^i^
		50	21.9 ^lm^
		10	10.0 ^qrst^
		1	0.0 ^w^
	72	100	47.0 ^e^
		50	31.9 ^h^
		10	19.6 ^mn^
		1	11.0 ^qrs^
Control	-	-	0.0 ^w^

Each value is the mean of five replicates; values in each column followed by the same in the latter are not significantly different according to Duncan’s multiple range test (DMRT) at *p* ≤ 0.05.

**Table 4:** Effect of different stages of stipe of *Ganoderma lucidum* on juvenile mortality of *Meloidogyne incognita*.

Age of Fungus	Exposure Period (h)	Concentration (%)	Juvenile Mortality (%)
Two week	12	100	23.8 ^j^
		50	16.4 ^n^
		10	9.1 ^qr^
		1	0.0 ^x^
	24	100	32.4 ^ef^
		50	19.5 ^lm^
		10	13.0 ^o^
		1	5.3 ^uv^
	48	100	47.8 ^c^
		50	28.8 ^gh^
		10	17.5 ^mn^
		1	6.7 ^tu^
	72	100	73.8 ^a^
		50	46.6 ^c^
		10	30.9 ^fg^
		1	16.9 ^n^
Four week	12	100	10.2 ^pqrs^
		50	3.4 ^vw^
		10	0.0 ^x^
		1	0.0 ^x^
	24	100	18.7 ^mn^
		50	10.4 ^pqr^
		10	4.0 ^vw^
		1	2.3 ^wx^
	48	100	33.6 ^e^
		50	25.3 ^ij^
		10	11.5 ^opq^
		1	4.1 ^vw^
	72	100	61.6 ^b^
		50	33.7 ^e^
		10	21.3 ^kl^
		1	13.0 ^o^
Eight week	12	100	0.0 ^x^
		50	0.0 ^x^
		10	0.0 ^x^
		1	0.0 ^x^
	24	100	12.2 ^op^
		50	7.1 ^tu^
		10	1.9 ^wx^
		1	0.0 ^x^
	48	100	27.4 ^hi^
		50	18.4 ^mn^
		10	7.8 ^st^
		1	0.0 ^x^
	72	100	38.9 ^d^
		50	23.3 ^jk^
		10	11.6 ^op^
		1	8.0 ^rst^
Control	-	-	0.0 ^x^

Each value is the mean of five replicates; values in each column followed by the same in the latter are not significantly different according to Duncan’s multiple range test (DMRT) at *p* ≤ 0.05.

**Table 5:** The major compounds detected in the ethanolic extract of *G. lucidum* parts (pileus and stipe) by GC-MS analysis.

Fungus Part	Peak Number	Retention Time (Min)	Area	Compound	Molecular Formula	Chemical Functional Group
Pileus	1	8.291	911,259	2,3-Dihydro- 3,5-Dihydroxy- 6-Methyl -(4H)-pyran-4-one	C_6_H_8_O_4_	Pyrones
	2	8.712	574,471	2-Hexene-3,4,4-Trimethyl	C_9_H_18_	Alkenes
	3	15.403	5,934,223	Beta-D-Glucopyranoside, methyl	C_7_H_14_O_6_	Glucosides
	4	16.504	83,965,777	DL-Arabinitol	C_5_H_12_O_5_	Sugar Alcohol
	5	19.792	66,792,284	D-Mannitol	C_6_H_14_O_6_	Sugar Alcohol
	6	28.708	6,731,054	Silane, dimethyl (3-fluorophenoxy) tetradecyloxy	C_22_H_39_FO_2_Si	Silanes
	7	29.946	10,642,382	Cholesta-8,14-dien-3-ol, 3 beta, 5-alpha	C_27_H_44_O	Steroids
Stipe	1	15.585	169,985	(3aR,4R,7R)-1,4,9,9-Tetramethyl-3,4,5,6,7,8-hexahydro-2H-3a,7-methanoazulen-2-one	C_5_H_22_O	Cyclic Ketones
	2	17.799	531,800	Hexadecanoic acid, methyl ester	C_17_H_34_O_2_	Palmitates
	3	19.428	3,143,212	9,12-Octadecadienoic acid (Z,Z)-, methyl ester	C_19_H_34_O_2_	Linoleic Acids
	4	19.487	5,395,870	9-Octadecenoic acid, methyl ester, (E)	C_19_H_36_O_2_	Oleic Acids
	5	19.725	173,129	Methyl stearate	C_19_H_38_O_2_	Ester
	6	23.178	454,082	Bis(2-ethylhexyl) phthalate	C_24_H_38_O_4_	Phthalic Acid

**Table 6:** Effect of *G. lucidum* CFs on the multiplication of *M. incognita* infesting eggplant.

Treatments	Juveniles/kg Soil	Females/Root System	Number of Galls/Root System	Number of Egg Masses/Root System	Number of Eggs/Egg Mass
100%	5293 ^e^	123 ^e^	68 ^e^	97 ^de^	129 ^f^
50%	6342 ^d^	153 ^d^	83 ^d^	103 ^d^	165 ^e^
10%	11,942 ^c^	259 ^bc^	126 ^bc^	131 ^bc^	330 ^bcd^
1%	12,349 ^b^	278 ^b^	132 ^ab^	137 ^ab^	342 ^bc^
UIC	12,844 ^a^	327 ^a^	139 ^a^	146 ^a^	355 ^a^
UUC	-	-	-	-	-

Each value is the mean of five replicates. Values in each column followed by the same in the latter are not significantly different according to Duncan’s multiple range test (DMRT) at *p* ≤ 0.05. UIC—untreated inoculated control; UUC—untreated uninoculated control.

**Table 7:** Effect of *G. lucidum* CFs on the growth and physiological parameters of eggplant in relation to *M. incognita*.

Treatment	Plant Length (cm)		Plant Fresh Weight (g)		Plant Dry Weight (g)		Physiological Parameters		Yield/Plant (g)
	Shoot	Root	Shoot	Root	Shoot	Root	Total Chlorophyll (mg/g Fresh Leaf)	Carotenoid (mg/g Fresh Leaf)	
100%	48.39 ^b^	18.86 ^b^	220 ^b^	92.0 ^b^	34.20 ^b^	10.45 ^b^	1.93 ^b^	0.81 ^b^	605 **^b^**
50%	46.98 ^bc^	18.52 ^b^	192 ^c^	79.5 ^c^	28.25 ^c^	9.10 ^c^	1.67 ^c^	0.69 ^c^	530 ^c^
10%	45.32 ^bc^	17.25 ^bc^	147 ^d^	61.0 ^d^	23.50 ^d^	7.40 ^d^	1.50 ^d^	0.60 ^cd^	432 ^d^
1%	43.56 ^cd^	16.99 ^cd^	132 ^e^	48.0 ^e^	22.10 ^de^	6.40 ^de^	1.49 ^de^	0.58 ^cd^	376 ^e^
UIC	25.45 ^e^	13.3 ^e^	110 ^f^	40.0 ^f^	22.63 ^de^	6.45 ^de^	1.45 ^d^	0.54 ^de^	347 ^ef^
UUC	54.26 ^a^	21.57 ^a^	267 ^a^	106.0 ^a^	46.33 ^a^	16.55 ^a^	2.72 ^a^	0.96 ^a^	876 ^a^

Each value is the mean of five replicates. Values in each column followed by the same in the latter are not significantly different according to Duncan’s multiple range test (DMRT) at *p* ≤ 0.05. UIC—untreated inoculated control; UUC—untreated uninoculated control.

## Data Availability

The data presented in this study are available within the article.
